# Baseline Occlusion Angiographic Appearance on Mechanical Thrombectomy Suggests Underlying Etiology and Outcome

**DOI:** 10.3389/fneur.2019.00499

**Published:** 2019-05-08

**Authors:** Pablo Garcia-Bermejo, Satya Narayana Patro, Ayman Z. Ahmed, Ghaya Al Rumaihi, Naveed Akhtar, Sadaat Kamran, Abdul Salam, Ahmed Own, Maher Saqqur, Ashfaq Shuaib

**Affiliations:** ^1^Neuroradiology Department, Hamad General Hospital, Hamad Medical Corporation, Doha, Qatar; ^2^Neurology Department, Hamad General Hospital, Hamad Medical Corporation, Doha, Qatar; ^3^Neurosurgery Department, Hamad General Hospital, Hamad Medical Corporation, Doha, Qatar; ^4^Neuroscience Institute, Hamad General Hospital, Hamad Medical Corporation, Doha, Qatar; ^5^Department of Medicine (Neurology), University of Alberta, Edmonton, AB, Canada

**Keywords:** mechanical thrombectomy, large vessel occlusion, intracranial atherosclerosis, acute ischemic stroke, occlusion appearance

## Abstract

**Background:** Etiology of a large vessel occlusion is relevant in the management of acute ischemic stroke patients and often difficult to determine in the acute phase.

**Aims:** We aim to investigate whether the angiographic appearance of the occlusion is related to its etiology and outcome.

**Materials and Methods:** Patients without cervical carotid occlusions who underwent mechanical thrombectomy in our center from April 2015 to September 2018 were studied. Demographics, clinical and radiological variables and outcome measures, including etiological classification of stroke, were collected. Underlying intracranial atherosclerosis was estimated according to the presence of stenosis after recanalization. Patients were assigned to groups based on the appearance of the occlusion observed in the first angiogram as “tapered” or “non-tapered.” Differences were searched amongst them.

**Results:** 131 patients met inclusion criteria. 31 (23.6%) were “tapered” and 100 (76.3%) non-tapered. Tapered presented lower mean baseline NIHSS (10.3 ± 6.2 vs. 16.1 ± 7.2; *p* < 0.001), smaller acute infarct cores as CTP CBV ASPECTS (8.6 ± 1.6 vs. 7.2 ± 2.4; *p* = 0.003), higher proportion of instant re-occlusions (26.7 vs. 8.2%; *p* = 0.025), fewer complete recanalization (45.2 vs. 71.0%; *p* = 0.028), and more persistent occlusions (37.5 vs. 10.6%; *p* = 0.011) on follow up MRA. There were no differences in reperfusion rates (83.9 vs. 84.0%; *p* = 0.986) nor in good long term functional outcome (50.0 vs. 51.1%; *p* = 0.921). Intracranial atherosclerosis etiology was more common in tapered than in non-tapered occlusions (54.8 vs. 18.0%; *p* < 0.001).

**Conclusion:** The angiographic appearance of an occlusion in mechanical thrombectomy patients may determine its etiology, predict likelihood of successful recanalization, and risk of reocclusion.

## Introduction

Endovascular Treatment with stent-retrievers has become a standard of care for acute ischemic stroke patients with large vessel occlusion (1). However, despite significant improvement, especially for revascularization rates, procedural times, and safety profile (2), near to 30% of patients experience failure to achieve reperfusion, which may lead to poor functional outcomes (3).

Several factors may interfere with early achievement of recanalization and good outcome, including difficult vessel anatomy, thrombus amount and characteristics, tandem occlusions, and the underlying etiology of the stroke (4). Acute intracranial atherosclerosis occlusions (ICAS-O) with *in-situ* thrombosis may be especially resistant to recanalization with conventional mechanical thrombectomy (MT) with stent-retrievers (5, 6), requiring the interventionists to consider additional strategies to maintain the patency of the occluded arterial segment (7). It is therefore important to study predictors of recanalization failure and to understand the etiology of the occlusion as soon as possible in planning the MT procedure for better outcome.

Based on this, the aim of our study was to investigate whether the initial appearance of the occlusion can help us to distinguish the nature of the underlying lesion and to predict radiological and clinical outcome.

## Methods

Qatar is a rapidly developing country with a population of 2.6 million, most of them expats with very diverse origins, the majority from the South-Asian subcontinent. Our center (Hamad General Hospital) is the main tertiary center in the country and the only one with a Comprehensive Stroke Center and an Interventional Neuroradiology Unit.

### Patients

Approximately 1200 stroke patients are admitted to our Hospital every year (8). Each patient is registered prospectively in a “stroke database” with an ancillary section for interventional cases. From this database, we collected patients consecutively selected for MT since the establishment of the Interventional Neuroradiology Unit in April 2015 until September 2018. In order to study the angiographic appearance of the occlusion, we excluded cases with recanalization on initial angiogram, who did not receive any EVT, and those with occlusion of the cervical ICA, which impedes visualization of the intracranial occlusion.

On arrival to the Emergency Department, all acute ischemic stroke patients are assessed immediately by the stroke team and undergo urgent multimodal imaging including non-contrast CT (NCCT) head scan, computed tomography angiography (CTA), and computed tomography perfusion (CTP). When patients are eligible for iv rtPA, the bolus is given in the CT room immediately after the NCCT and prior to the performance of the CTP and CTA.

### Clinical Variables

Baseline demographics were studied including age, gender, NIHSS, risk factors for atherosclerosis (hypertension, diabetes mellitus, dyslipidemia, smoking, previous TIA), atrial fibrillation (AF), and congestive heart failure (CHF).

Good functional outcome was defined as a mRS < 3 at 90 days. It was obtained in a follow up clinic visit by a mRS certified neurologist or by telephone interview when patients couldn't attend the clinic.

### Neuroimaging Studies

Radiological variables were assessed by two readers. ASPECT score was used to calculate the early ischemic changes. Intracranial hyperdense artery sign on NCCT was collected independently of its location (ICA, M1, M2, or vertebrobasilar).

Patients undergo a 24 h follow up NCCT, followed up by a MRI-MRA examination usually between 24 and 72 h. Symptomatic hemorrhagic transformation was defined according to ECASS III criteria (9). The status of the arterial occlusion on MRA was categorized as: “persistent recanalization,” “partial recanalization” when any degree of stenosis was present, or “persistent occlusion.”

### Angiographic Appearance of the Arterial Occlusion

**Figure 1:**
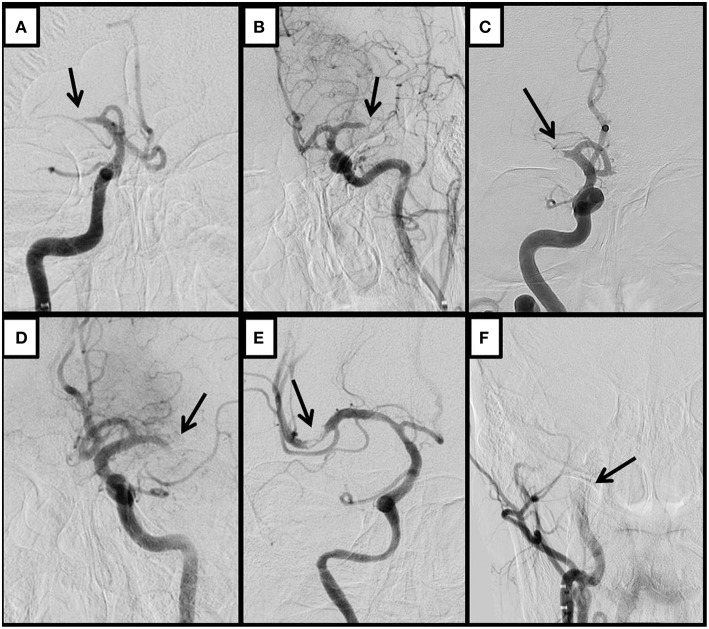
Representative cases of tapered **(A,B)**, meniscus **(C)**, cutoff **(D)**, Tramtrack **(E)**, and Undetermined occlusions. The initial angiogram shows the appearance of the occlusion. An arrow indicates the occlusive lesion. Tapered occlusions are shown in the superior row **(A,B)**; **(A)** Right ICA run shows gradual narrowing of the lumen of the proximal M1 segment of the middle cerebral artery (MCA) ending with an acute angle over the superior wall of the vessel; **(B)** Angiogram from the left ICA shows similar pattern of gradual narrowing although with the acute angulation over the inferior wall of the distal M1 segment of the left MCA; **(C)** Right ICA angiogram shows a proximal M1 occlusion of the right MCA, with an abrupt blockage of the artery depicting a concavity toward the proximal lumen, representing a meniscus occlusion; **(D)** Left ICA run shows a distal M1 occlusion, where contrast ends abruptly without delineating a specific shape, displaying a cutoff occlusion; **(E)** Right ICA angiogram shows a partial occlusive lesion at the proximal superior M2 segment of the right MCA, with distal contrast filling that allows visualizing several pieces of thrombi material in line, representing a tram-track occlusion; **(F)** Proximal right ICA run shows stagnation of contrast through the ascending carotid artery impending the visualization of an occlusive lesion that is located more distal to the reach of the contrast, depicting therefore an Undetermined occlusion.

**Figure 2:**
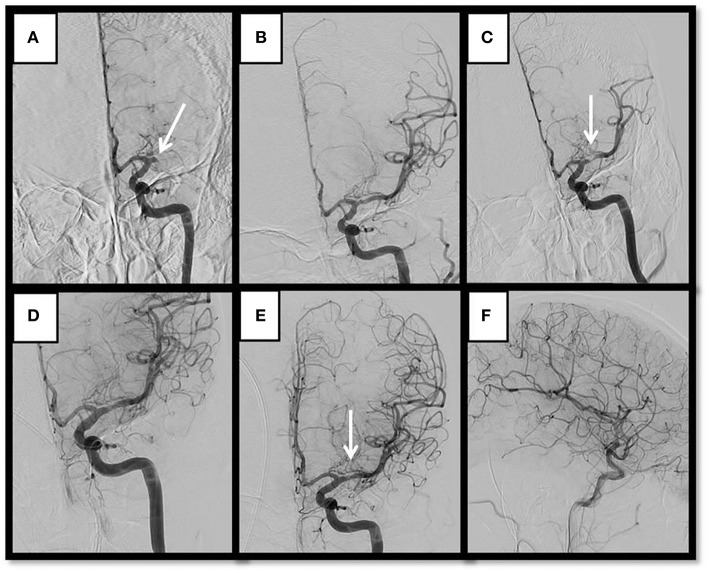
Representative MT of a case with a tapered occlusion who experienced severe stenosis after pass and required rescue therapy with permanent stenting and GP IIb/IIIa inhibitors. Thirty-six year-old Bangladeshi male with history of HTN, presenting a “wake up” complete left MCA syndrome (NIHSS 20). **(A)** Baseline angiogram shows left proximal M1 occlusion with tapered aspect. **(B)** Complete recanalization after 1 pass of stent-retriever. **(C)** Early spontaneous severe re-stenosis after 10 min. **(D)** By pass of detachable stent-retriever over the lesion after IA bolus of eptifibatide. **(E,F)** Anteroposterior and lateral views of complete recanalization after detachment of stent showing complete recanalization and reperfusion.

A classification of the angiographic appearance of intracranial occlusions was adapted (10) for our study. We choose the first DSA angiogram to classify its appearance (Figure 1). We hypothesized that the appearance may be related with the underlying etiology of the lesion, specifically that tapered occlusions represents ICAS and therefore are more resistant to recanalize with the standard stent-retriever techniques (Figure 2). On the contrary, the rest of the occlusion types might be more likely related with embolic sources.

Two neurointerventionists retrogradely reviewed the initial angiograms blinded to the outcome, and classified the appearance of the occlusions. In cases with disagreement the final morphologic designation was established by consensus. The kappa value for the interrater reliability was 0.68 (substantial agreement).

### Mechanical Thrombectomy

The only technique used was MT with stent-retriever, either with Solitaire FR or AB, or Trevo XP. The use of balloon guide catheter occurred at the discretion of the neuro-interventionist performing the procedure.

During the performance of the procedure, we examined the occurrence of: good collaterals on DSA (reaching more than half of the territory of the artery occluded at the baseline run), stenosis of the Target Arterial Occlusive Lesion (AOL) immediately after any of the passes with the stent-retriever, instant reocclusion of a lesion previously recanalized, and the total number of passes until recanalization or end of the procedure.

We classified the type of occlusion as “truncal” (all major branches and its bifurcation site was clearly visible beyond the occlusion segment after achieving minimal recanalization of the original occlusion) or “branching” (another branch could not be seen or was only partially seen when the stent-retriever was deployed up to one branch across the occlusion site) (11). Truncal occlusion type is considered an indicative of ICAS-O.

According to literature from countries with high prevalence of ICAS-O, additional rescue therapy was administered in some. It consisted of the use of a parenteral antiplatelet agent alone (Eptifibatide) and/or permanent stent detachment over the occlusive lesion (Solitaire AB) (12, 13).

Angiographic outcome endpoints were final reperfusion assessed with the mTICI score (full reperfusion was defined as mTICI 2b-3), and recanalization as per the AOL score (0: no recanalization of the primary AOL; 1: complete or partial recanalization of the primary occlusive lesion with no distal flow; 2: incomplete or partial recanalization of the primary occlusive lesion with any distal flow; 3: complete recanalization of AOL with any distal flow) (14). Cases with AOL 3 were categorized as “complete recanalization,” AOL 2 “partial recanalization,” and AOL 0-1 “complete occlusion.”

### ICAS-0 and Etiologic Classification of Stroke

ICAS-O was defined according to the presence of any degree of stenosis in the final angiogram post-recanalization.

The classification of other stroke etiologies was based on the TOAST criteria (15) and performed by a stroke neurologist and an interventional neurologist. The final etiology was classified into three groups: ICAS, Cardioembolic, and Undetermined, as patients with proximal ICA occlusion were excluded from the study. Undetermined group includes all those patients with undetermined cryptogenic, incomplete etiological investigation, and both Cardioembolic and ICAS criteria.

### Statistical Analysis

All statistical analyses were performed using Statistical Package for Social Sciences (SPSS) software (version 23.0, SPSS Inc. Chicago, Ill. USA). Descriptive and inferential statistics were used to characterize the study sample and test hypotheses. Descriptive results (including graphical displays) for all quantitative variables (e.g., age) are presented as mean ± standard deviation (SD) (for normally distributed data), while numbers (percentage) were reported for all qualitative variables (e.g., gender). Bivariate analysis was performed using Independent Sample *t*-test or Mann Whitney *U*-test whenever appropriate to compare the average for all quantitative variables (e.g., age) between tapered vs. non-tapered, while Pearson Chi-Square test or Fisher Exact test as appropriate were used to compare all the qualitative variables (e.g., gender) between tapered vs. non-tapered. A “*P*” value < 0.05 (two tailed) was considered statistically significant.

## Results

During the study period 169 patients were selected for MT. Fifteen of them presented recanalization on the first run and were not treated, four patients showed occlusions of the proximal ICA only, 17 had tandem cervical ICA-intracranial occlusions, and two cases were lost leaving 131 patients for inclusion in the study (mean age 50. 9 ± 13.9; M:F = 107:24). Mean NIHSS was 14.7 ± 7.2, mean CT ASPECTS 8.9 ± 1.4. One hundred and ten (84.0%) patients experienced successful reperfusion, after a mean number of passes of 1.9 ± 1.4 in the whole sample. Lastly 57 (50.9%) patients presented with good functional outcome at 90 days (19 patients missed follow up). The etiology of the stroke was classified as ICAS in 35 (26.7%), Cardioembolic in 46 (35.1%), and Undetermined in 50 (38.2%) of the cases.

Patients were assigned into five groups according to the appearance of their occlusion as follows: 31 (23.7%) Tapered, 36 (27.5%) Meniscus, 44 (33.6%) Cutoff, 5 (3.8%) Tram-track, and 15 (11.5%) were Undetermined. Characteristics and Differences amongst these groups are shown in the Supplementary Material.

When comparing patients with tapered vs. non-tapered occlusions, we found that patients with tapered occlusions presented a higher proportion of ICAS (54.8 vs. 18.0%), and lower proportion of Cardioembolic (16.1 vs. 41.0%) (*p* < 0.001) (Table 1). The tapered group showed smaller acute infarct cores at baseline as measured with mean ASPECTS on CTP CBV (8.6 ± 1.6 vs. 7.2 ± 2.2; *p* = 0.003).

**Table 1:** Comparison of baseline demographics, etiological classification of stroke and clinical outcome between tapered and non-tapered occlusion groups.

	**Overall (*n* = 131)**	**Tapered (*n* = 31)**	**Non-tapered (*n* = 100)**	***P*-value**
Age	50.9 ± 13.9	50.6 ± 12.6	50.9 ± 14.3	0.901
**Sex**				
• Male	107(81.7%)	26(83.9%)	81(81.0%)	0.718
Baseline NIHSS	14.7 ± 7.2	10.3 ± 6.2	16.1 ± 7.0	< 0.001
**Risk Factors**				
• HTN	77(58.8%)	20(64.5%)	57(57.0%)	0.458
• DM	53(40.5%)	13(41.9%)	40(40.0%)	0.848
• Dyslipidemia	29(23.0%)	6(20.0%)	23(24.0%)	0.653
• AF	26(23.9%)	4(16.0%)	22(26.2%)	0.294
• CHF	7(5.3%)	0(0.0%)	7(7.0%)	0.130
• TIA	10(7.6%)	2(6.5%)	8(8.0%)	0.777
• Smoking	18(13.7%)	4(12.9%)	14(14.0%)	0.877
Thrombolysis given	78(59.5%)	15(48.4%)	63(63.0%)	0.148
TOAST Classification				< 0.001
• ICAS	35(26.7%)	17(54.8%)	18(18.0%)	
• Cardioembolic	46(35.1%)	5(16.1%)	41(41.0%)	
• Undetermined	50(38.2%)	9(29.0%)	41(41.0%)	
Good long term outcome ≤ 2^(n = 112)^	57(50.9%)	12(50.0%)	45(51.1%)	0.921

During the intervention, the tapered group presented a higher rate of truncal type occlusions (76.9 vs. 31.1%; *p* < 0.001), stenosis immediately after a pass of stent-retriever (85.2 vs. 31.3%; *p* < 0.001), and resulted with a higher proportion of patients with instant re-occlusions (26.7 vs. 8.2%; *p* = 0.025). Accordingly, the use of additional rescue therapy with permanent stenting was more frequent in the tapered group (51.6 vs. 14.1%; *p* ≤ 0.001). There were no differences in the final reperfusion rates amongst these groups (83.9 vs. 84.0%; *p* = 0.986) (Table 2).

**Table 2:** Comparison of imaging variables and interventional performance between tapered and non-tapered groups.

	**Overall (*n* = 131)**	**Tapered (*n* = 31)**	**Non-tapered (*n* = 100)**	***P*-value**
NCCT ASPECTS	8.9 ± 1.4	9.1 ± 0.9	8.8 ± 1.5	0.504
CTP CBV ASPECTS	7.5 ± 2.2	8.6 ± 1.6	7.2 ± 2.2	0.003
Hyperdense sign NCCT	65(50.4%)	8(25.8%)	57(57.0%)	0.002
Concomitant stenosis non-target vessel	21(19.3%)	12(44.4%)	9(11.0%)	< 0.001
Proximal vessel wall irregularity	37(28.2%)	19(61.3%)	18(18.0%)	< 0.001
Angiographic occlusion site				0.016
• Distal ICA	21(16.0%)	2(6.5%)	19(19.0%)	
• M1	75(57.3%)	25(80.6%)	50(50.0%)	
• M2	19(14.5%)	3(9.7%)	16(16.0%)	
• ACA	1(0.8%)	0(0.0%)	1(1.0)	
• Vertebrobasilar system	14(10.7%)	1(3.2%)	13(13.0%)	
Good collaterals on DSA	63(67.7%)	20(74.1%)	43(65.2%)	0.403
Occlusion type^(n = 116)^				< 0.001
• Truncal	48(41.4%)	20(76.9%)	28(31.1%)	
• Branching	68(58.6%)	6(23.1%)	62(68.9%)	
Stenosis after pass of stent-retriever^(n = 123)^	53(43.1%)	23(85.2%)	30(31.3%)	< 0.001
Intraprocedural reocclusion^(n = 127)^	16(12.6%)	8(26.7%)	8(8.2%)	0.025
Use of additional rescue therapy	30(23.1%)	16(51.6%.)	14(14.1%)	< 0.001
Parenteral antiplatelet agent	26(20.0%)	14(45.2%)	12(12.1%)	0.001
Stent detachment	25(19.1%)	15(48.4%)	10(10.0%)	< 0.001
Number of passes	1.9 ± 1.4	1.6 ± 0.9	2.0 ± 1.5	0.132
Reperfusion (mTICI 2b/3)	110(84.0%)	26(83.9%)	84(84.0%)	0.986
Symptomatic ICH	6(5.3%)	0(0.0%)	6(6.7%)	0.191

The outcome of the occlusive lesion was different between both groups. Immediately after the procedure, the tapered group presented higher proportion of cases with residual stenosis (41.9 vs. 17.0%; *p* = 0.028). Moreover, on follow up MRA, we found a higher rates of persistent occlusions (37.5 vs. 10.6%) with a significantly lower rates of persistent full recanalization (41.7 vs. 66.7%; *p* = 0.011) in patients with tapered occlusions (Table 3).

**Table 3:** Comparison of arterial occlusive lesion outcome between tapered and non-tapered groups.

	**Overall (*n* = 131)**	**Tapered (*n* = 31)**	**Non-tapered (*n* = 100)**	***P*-value**
Post-recanalization Target Artery status				0.028
• Complete occlusion	16(12.2%)	4(12.9%)	12(12.0%)	
• Partial recanalization	30(22.9%)	13(41.9%)	17(17.0%)	
• Complete recanalization	85(64.9%)	14(45.2%)	71(71.0%)	
Follow up MRA Target Artery status^(n = 90)^				0.011
• Persistent occlusion	16(13.3%)	9(37.5%)	7(10.6%)	
• Partial recanalization	20(16.6%)	5(20.8%)	15(22.7%)	
• Complete recanalization	54(45.0%)	10(41.7%)	44(66.7%)	

Symptomatic ICH occurred less frequently in the tapered occlusion group, however the difference was not significant (0 vs. 6.7%; *p* = 0.191).

## Discussion

Our study shows that the angiographic appearance of a large vessel occlusion in patients with acute ischemic stroke secondary to ICAS has characteristic appearance that can help differentiate it from suspected embolic mechanisms. Accordingly, the baseline appearance of the occlusion helps predicting resistance to recanalization, allowing the interventionist to prepare a specific MT strategy in order to facilitate early and complete recanalization.

Our observations during MT shows that tapered lesions had a different behavior during the procedure than other type of occlusions. Firstly, there is a higher frequency of early re-occlusions during the procedure, residual stenosis and, perhaps even more deleterious, more frequent delayed re-occlusions detected in the follow up MRA despite satisfactory recanalization after the MT. Moreover, we suggest that it is reasonable to suspect a “harder than a clot” underlying occlusive lesion when its angiographic edge is sharper, instead of dull (like in the meniscus or cut-off occlusions), assuming that the consistency of the occlusive material could be related to its appearance. In addition, such lesions are significantly more likely to need additional interventional measures to try to maintain the patency of the artery. These additional measures include the use of parenteral antiplatelet agents alone or in combination with permanent stenting.

Our “rescue therapy approach” is in agreement with the literature from countries with high rates of ICAS population and refractory occlusions (12, 13, 16–18). In a study with 318 stroke patients treated with MT, Bhaek et al. found 17.6% truncal type occlusions, defined by them as ICAS-O. These cases were less likely to recanalize with the standard stent-retriever technique alone (only 28.9%), and required more frequently additional rescue therapy to reach a similar rate of reperfusion (80.4 vs. 88.5%; *p* = 0.097) and good long term functional outcome (46.4% vs. 46.9; *p* = 0.944) to the non-ICAS-O group (13). These results are similar to those found in our study. However, the identification of ICAS-O by means the angiographic appearance of the occlusion, could be in our opinion, simpler and faster. It requires only the baseline angiogram of the procedure, whereas to determine the type of occlusion it is necessary to assess the distribution of the flow beyond the occlusive lesion, either through the anterior communicating artery in distal ICA occlusions or through the by-pass of the stent-retriever once it is deployed covering the occlusion.

In our study sample, patients in whom tapered occlusion lesions were evident also had additional angiographic markers of atherosclerosis, including more frequent truncal type occlusions, concomitant stenosis in other vessels, proximal angiographic irregular contours on DSA, and residual stenosis after recanalization. Similar lesions were less likely to be evident in other types of occlusion.

In addition we noticed that patients with tapered occlusions presented to the emergency department with milder symptoms and smaller infarct sizes on neuroimaging. This is typical of patients with acute ischemic stroke large vessel occlusions due to ICAS (19). It has been argued that the progressive growth of the atherosclerotic plaque allows individuals to gradually develop collaterals in response to the consequent decrease in the distal perfusion pressure (20). Moreover, the high rate of stenosis after pass as was evident in our patients with large vessel occlusion secondary to ICAS is in agreement with the assumption that ICAS occlusions recanalize in a stepwise manner compared with embolisms, which are more likely to recanalize in an “all or nothing” way. This has been previously documented in the literature attributed to ICAS occlusions (19), which has consequently led to an increased use of rescue therapy (12, 15, 16, 18). In agreement with this, the hyperdense artery sign on NCCT was less frequent in the tapered group. Although it has not been clearly associated with a particular pathogenesis of the arterial occlusion, it is well-recognized that it is produced by the signal of the intra-luminal thrombosis and has been associated with more severe strokes and poorer outcomes, probably because it reflects a larger amount of clot inside the vessel (21–23). Thus, lesions with a higher plaque/thrombus ratio may be less likely to present this sign, as tapered occlusions, which supports our hypothesis of more frequent underlying ICAS in this group.

Interestingly, besides the highest rate of immediate post-recanalization residual stenosis, the tapered group had the highest proportion of follow up occlusions on MRA, despite more frequent use of rescue therapy with permanent stenting. Thus, tapered occlusions, despite satisfactory reperfusion after the MT, were at a higher risk of further reocclusion and perhaps clinical deterioration, although further research is needed to confirm its symptomatic correlation. The observation that patients with underlying ICAS-O have higher rate of early and delayed reocclusions with an impact on the clinical outcome has already been reported (19).

In our study, the long term functional outcome was very similar in all groups of patients, even though the baseline stroke severity and the size of the early infarction were smaller in the tapered group. The relatively poor prognosis in the ICAS patients despite the smaller size of ischemic strokes, we believe, may be related to the increased incidence of reocclusions in this group of patients (19). Our sample size may however be too small to provide an accurate answer to this question.

Our study has limitations. First of all, our patient population is a peculiar, mainly young, male, and multi-ethnical sample, primarily from Arabic and South and East Asian countries. Thus, we are not sure whether our results can be extrapolated to other groups of people. Our sample size is limited, making it difficult to adjust our results for cofounders. Moreover, the follow up MRA was not performed in some patients, what makes it difficult to assume strong conclusions.

Our data suggests that an angiographic tapered appearance of the intracranial occlusion on acute ischemic stroke patients selected for MT may reflect an underlying ICAS-O and therefore a poorer angiographic outcome. This may represent a novel angiographic marker that may allow interventionist to early predict refractoriness and prepare an appropriate intervention strategy accordingly. However, further multi-center studies with a larger sample size and a population with different ethnicities or origins are warranted prior to being able to extrapolate our results to different contexts.

## Ethics Statement

The study is observational. Data was anonymized and obtained from a prospective registry approved by our institutional IRB.

## Author Contributions

Both MS and AsS contributed equally as senior authors. PG-B conceived the hypothesis, extracted the data, performed the analysis, and wrote the manuscript. SP categorized the angiographic variables with PG-B. SK extracted radiological and data and classified the etiology with PG-B. AbS supported with the statistical analysis. NA and AA supported the conceptual design of the study. GA and AO supported the research and contributed substantially to data acquisition. All co-authors revised and approved the manuscript.

### Conflict of Interest Statement

The authors declare that the research was conducted in the absence of any commercial or financial relationships that could be construed as a potential conflict of interest.
