# Supplementary material for: Baseline Occlusion Angiographic Appearance on Mechanical Thrombectomy Suggests Underlying Etiology and Outcome
Source: Front Neurol. 2019 May 8;10:499. doi: 10.3389/fneur.2019.00499 (PMC6517505; doi:10.3389/fneur.2019.00499)
Supplement: Supplementary file 1 [file Data_Sheet_1.docx]

| Variable | Tapered  (n=31) | Meniscus (n=36) | Cut off  (n=44) | Tramtrack (n=5) | Undetermined (n=15) | P value |
| --- | --- | --- | --- | --- | --- | --- |
| Age | 50.6 ± 12.6 | 53.8 ± 13.6 | 49.5 ±15.7 | 37.4 ± 7.8 | 52.8 ± 10.7 | 0.075 |
| Sex |  |  |  |  |  |  |
| - Male | 126 (83.9%) | 28 (77.8%) | 36 (81.8%) | 4 (80.0%) | 13 (86.7%) | 0.948 |
| Baseline NIHSS | 10.3 ± 6.2 | 16.4 ± 8.5 | 16.5 ± 5.5 | 12.2 ± 9.9 | 16.3 ± 6.2 | 0.002 |
| Risk factors |  |  |  |  |  |  |
| - HTN | 20 (64.5%) | 24 (66.7%) | 21 (47.7%) | 3 (60.0%) | 9 (60.0%) | 0.466 |
| - DM | 13 (41.9%) | 14 (38.9%) | 17 (38.6%) | 3 (60.0%) | 6 (40.0%) | 0.922 |
| - DL | 6 (20.0%) | 8 (23.5%) | 8 (19.0%) | 3 (60.0%) | 4 (26.7%) | 0.342 |
| - AF | 1 (3.2%) | 7 (19.4%) | 8 (18.2%) | 0 (0.0%) | 3 (20.0%) | 0.233 |
| - CHF | 0 (0.0%) | 1 (2.8%) | 4 (9.1%) | 0 (0.0%) | 2 (13.3%) | 0.230 |
| - TIA | 2 (6.5%) | 1 (2.8%) | 5 (11.4%) | 1 (20%) | 1 (6.7%) | 0.519 |
| - Smoking | 4 (12.9%) | 3 (8.3%) | 7 (15.9%) | 2 (40.0%) | 2 (13.3%) | 0.407 |
| Thrombolysis given | 15 (48.4%) | 26 (72.2%) | 24 (54.5%) | 2 (40.0%) | 11 (73.3%) | 0.169 |
| TOAST Classification |  |  |  |  |  | 0.001 |
| - ICAS | 17 (54.8%) | 3 (8.7%) | 12 (27.3%) | 0 (0.0%) | 3 (20.0%) |  |
| - Cardioembolic | 5 (16.1%) | 19 (52.8%) | 16 (36.4%) | 1 (20.0%) | 5 (33.3%) |  |
| - Undetermined | 9 (29.0%) | 14 (38.9%) | 16 (36.4%) | 4 (80.0%) | 7 (46.7%) |  |
| Good long term outcome <=2^(n=112)^ | 12 (50.0%) | 15 (48.4%) | 20 (51.3%) | 3 (75%) | 7 (50.0%) | 0.906 |

Supplementary material.

Table 1. Comparison of baseline demographics, etiological classification of stroke and clinical outcome amongst all angiographic appearance of occlusion groups.

Table 2. Comparison of imaging variables and interventional performance amongst all angiographic appearance of occlusion groups.

|  | Tapered  (n=31) | | Meniscus (n=36) | Cut off  (n=44) | Tramtrack (n=5) | Undetermined (n=15) | P value |
| --- | --- | --- | --- | --- | --- | --- | --- |
| NCCT ASPECTS | 9.1 ± 0.9 | | 9.1 ± 0.9 | 8.6 ± 1.8 | 9.2 ± 1.3 | 8.9 ± 1.8 | 0.779 |
| CTP CBV ASPECTS | 8.6 ± 1.6 | | 7.2 ± 1.9 | 7.1 ± 2.2 | 9.2 ± 1.5 | 6.7 ± 3.0 | 0.001 |
| Hyperdense sign NCCT | 8 (25.8%) | | 23 (63.9%) | 23 (54.8%) | 1 (20.0%) | 10 (66.7%) | 0.008 |
| Concomitant stenosis non-target vessel | 12 (44.4%) | | 2 (6.7%) | 5 (14.3%) | 0 (0.0%) | 2 (16.7%) | 0.003 |
| Proximal vessel wall irregularity | 19 (61.3%) | | 4 (11.1%) | 10 (22.7%) | 0 (0.0%) | 4 (26.7%) | < 0.001 |
| Angiographic occlusion site |  | |  |  |  |  | < 0.001 |
| - Distal ICA | 2 (6.5%) | | 5 (13.9%) | 1 (2.3%) | 1 (20.0%) | 12 (80.0%) |  |
| - M1 | 25(80.6%) | | 20 (55.6%) | 26 (59.1) | 2 (40.0%) | 2 (13.4) |  |
| - M2 | 3 (9.7%) | | 8 (22.2%) | 7 (15.9%) | 1 (20.0%) | 0 (0.0%) |  |
| - ACA | 0 (0.0%) | | 0 (0.0%) | 1 (2.3%) | 0 (0.0%) | 0 (0.0%) |  |
| - Vertebrobasilar system | 1 (3.2%) | | 3 (8.4%) | 9 (20.5%) | 0 (0.0%) | 1 (6.7%) |  |
| Good collaterals on DSA^(N=93)^ | 20 (74.1%) | | 17 (60.7%) | 23 (67.6%) | 1 (50%) | 2 (100%) | 0.668 |
| Occlusion type^(n=116)^ |  | |  |  |  |  |  |
| - Truncal | 20 (70.6%) | | 10 (30.3%) | 11 (29.7%) | 4 (80.0%) | 3 (20.0%) | <0.001 |
| - Branching | 6 (23.1%) | | 23 (69.7%) | 26 (70.3%) | 1 (20.0%) | 12 (80.0%) | <0.001 |
| Stenosis after pass of stent-retriever^(n=123)^ | 23 (85.2%) | | 8 (24.2%) | 15 (34.9%) | 2 (40.0%) | 5 (33.3%) | <0.001 |
| Intraprocedural reocclusion^(n=127)^ | 8 (26.7%) | | 2 (5.9%) | 4 (9.3%) | 1 (20.0%) | 1 (6.7%) | 0.177 |
| Use of additional rescue therapy | 16 (51.6%) | | 4 (11.4%) | 6 (13.6%) | 2 (40.0%) | 2 (13.3%) | <0.001 |
| Parenteral antiplatelet agent | 14 (45.2%) | | 3 (8.6%) | 6 (13.6%) | 2 (40.0%) | 1 (6.7%) | 0.009 |
| Stent detachment | 15 (48.4%) | | 2 (5.6%) | 4 (9.1%) | 2 (40.0%) | 2 (13.3%) | <0.001 |
| Number of passes | 1.6 ± 0.9 | | 2.1 ± 1.6 | 2.0 ± 1.5 | 2.6 ± 1.1 | 1.9 ± 1.4 | 0.151 |
| Reperfusion (mTICI 2b-3) | 26 (83.9%) | | 26 (72.2%) | 41 (93.2%) | 5 (100%) | 12 (80.0%) | 0.108 |
| Symptomatic ICH | 0 (0.0%) | 2 (6.5%) | | 3 (7.3%) | 0 (0.0%) | 1 (7.7%) | 0.713 |

Table 3. Comparison of Arterial Occlusive Lesion outcome amongst all angiographic appearance of occlusion groups.

|  | Tapered  (n=31) | Meniscus (n=36) | Cut off  (n=44) | Tramtrack (n=5) | Undetermined (n=15) | P value |
| --- | --- | --- | --- | --- | --- | --- |
| Post-procedure Target Artery Status ^(n=131)^   - Complete occlusion - Partial recanalization - Complete recanalization | 4 (12.9%)  13 (41.9%)  14 (45.2%) | 8 (22.2%)  3 (8.3%)  25 (69.4%) | 2 (4.5%)  11 (25.0%)  31 (70.5%) | 0  0  5 (100%) | 2 (13.3%)  3 (20.0%)  10 (66.7%) | 0.019 |
| Follow up MRA Target Artery Status ^(n=90)^   - Persistent occlusion - Partial recanalization - Complete recanalization | 9 (37.5%)  5 (20.8%)  10 (41.7%) | 4 (18.2%)  3 (13.6%)  15 (68.2%) | 3 (9.4%)  9 (28.1%)  20 (62.5%) | 0 (0.0%)  0 (0.0%)  4 (100%) | 0 (0.0%)  3 (37.5%)  5 (62.5%) | 0.073 |
